# Association study of stuttering candidate genes GNPTAB, GNPTG and NAGPA with dyslexia in Chinese population

**DOI:** 10.1186/s12863-015-0172-5

**Published:** 2015-02-03

**Authors:** Huan Chen, Junquan Xu, Yuxi Zhou, Yong Gao, Guoqing Wang, Jiguang Xia, Michael SY Huen, Wai Ting Siok, Yuyang Jiang, Li Hai Tan, Yimin Sun

**Affiliations:** State Key Laboratory of Proteomics, Beijing Proteome Research Center, Beijing Institute of Radiation Medicine, Beijing, 102206 China; National Engineering Research Center for Beijing Biochip Technology, Beijing, 102206 China; CapitalBio Corporation, Beijing, 102206 China; Department of Anatomy, The University of Hong Kong, Hong Kong, China; State Key Laboratory of Brain and Cognitive Sciences, The University of Hong Kong, Hong Kong, China; School of Humanities, The University of Hong Kong, Hong Kong, China; The State Key Laboratory Breeding Base-Shenzhen Key Laboratory of Chemical Biology, The Graduate School at Shenzhen, Tsinghua University, Shenzhen, China; Neuroimaging Laboratory, Department of Biomedical Engineering, School of Medicine, Shenzhen University, Shenzhen, China; Guangdong Key Laboratory of Biomedical Information Detection and Ultrasound Imaging, Shenzhen, 518060 China; Medical Systems Biology Research Center, Department of Biomedical Engineering, Tsinghua University School of Medicine, Beijing, China

**Keywords:** Developmental dyslexia, GNPTAB, GNPTG, NAGPA, SNPs

## Abstract

**Background:**

Dyslexia is a polygenic speech and language disorder characterized by an unexpected difficulty in reading in children and adults despite normal intelligence and schooling. Increasing evidence reveals that different speech and language disorders could share common genetic factors. As previous study reported association of *GNPTAB*, *GNPTG* and *NAGPA* with stuttering, we investigated these genes with dyslexia through association analysis.

**Results:**

The study was carried out in an unrelated Chinese cohort with 502 dyslexic individuals and 522 healthy controls. In all, 21 Tag SNPs covering *GNPTAB*, *GNPTG* and *NAGPA* were subjected to genotyping. Association analysis was performed on all SNPs. Significant association of rs17031962 in *GNPTAB* and rs882294 in *NAGPA* with developmental dyslexia was identified after FDR correction for multiple comparisons.

**Conclusion:**

Our results revealed that the stuttering risk genes *GNPTAB* and *NAGPA* might also associate with developmental dyslexia in the Chinese population.

**Electronic supplementary material:**

The online version of this article (doi:10.1186/s12863-015-0172-5) contains supplementary material, which is available to authorized users.

## Background

Speech and language disorders can be classified into numerous categories, including stuttering, speech sound disorder (SSD), verbal dyspraxia, specific language impairment (SLI) and developmental dyslexia (DD) [1]. Dyslexia, also known as reading disability (RD), is characterized by difficulties in reading and spelling despite of normal intelligence and adequate education background without any neurological impairments [2,3]. Though language disorders such as dyslexia are quite different concept from speech disorders, in many cases, it is difficult to discriminate a language disorder from a speech disorder in a specific individual [4]. Hence, some researchers regard them as a continuum of language disorders [5-7]. Motor deficiency might be one of the underlying mechanisms that explain how the two defects are connected. For instance, stuttering has been attributed to a temporal motor defect in speech preparation [8,9]. In terms of dyslexia, some recent studies have revealed that dyslexic individuals suffer from motor problems as well, especially in performing fine movements [6,10]. A great deal of evidence reveals that language disorders and speech disorders could share some genetic factors. For example, forkhead box P2 (*FOXP2*) and its downstream target gene contactin associated protein-like 2 (*CNTNAP2*) have been shown to be an important link in the networks of several speech and language disorders, including SLI, dyslexia, stuttering and dyspraxia [1,11-20]. This viewpoint triggered us to verify whether candidate genes for stuttering were also involved in the pathogenesis of developmental dyslexia.

Recently, in a study of stuttering individuals from Pakistan and North America, candidate gene and linkage analyses identified several mutations in the lysosomal enzyme-targeting pathway genes N-acetylglucosamine-1-phosphate transferase gene (*GNPTAB*), N-acetylglucosamine-1-phosphate transferase, gamma subunit (*GNPTG*) and N-acetylglucosamine-1-phosphodiester alpha-N-acetylglucosaminidase (*NAGPA*) [21]. Subsequent studies of stuttering identified mutations in the *GNPTAB* gene and two functionally related *GNPTG* and *NAGPA* genes in large families and in the sporadic patients, reaffirming their association with stuttering [22-24]. However, the relevance of these genes with dyslexia has not yet been reported. It has been shown that stuttering is more common in children who suffer from concomitant speech, language, or motor deficiencies, implying that speech and language disorders may be connected genetically to some extent. Therefore, the three genes (*GNPTAB*, *GNPTG* and *NAGPA*) that may predispose people to stuttering are potential candidate risk genes for other speech and language disorders. Based on the above evidence, we performed association analysis on these genes with dyslexia in a large unrelated Chinese cohort.

## Results

### Single marker analysis

In the present study, we performed genotyping on Tag SNPs of three candidate genes for stuttering, *GNPTAB*, *GNPTG* and *NAGPA*. Data adjustment for age and sex was performed on genotyping results. Table 1 shows the SNP markers with significant unadjusted p-values (<0.05) in the study.

**Table 1:** **Association between significant SNP markers and dyslexia using the additive, dominant, genotype, and the recessive models**

**Gene**	**SNP**	**Patient**	**Control**	**Crude OR (95%CI)**	**Unadjusted p-value**	**Adjusted OR (95%CI)**	**Adjusted p-value**	**FDR corrected p-value**
GNPTAB	rs17031962							
	C Allele	677	678	1.000		1.000		
	T Allele	287	340	0.844	0.082	0.748	0.006	0.065
				(0.6977-1.022)		(0.6079-0.9209)		
	CC	240	222	1.000		1.000		
	CT	197	234	0.779	0.062	0.674	0.007	0.074
				(0.5986-1.0131)		(0.5057-0.8977)		
	TT	45	53	0.785	0.279	0.672	0.093	0.326
				(0.5072-1.2161)		(0.4226-1.0687)		
	Dom			0.780	0.052	0.665	0.003	0.036
				(0.6074-1.002)		(0.5056-0.8739)		
	Rec			0.886	0.571	0.771	0.255	0.596
				(0.5831-1.346)		(0.4917-1.207)		
	rs10778148							
	C Allele	854	909	1.000		1.000		
	T Allele	110	107	1.090	0.540	1.148	0.368	0.639
				(0.827-1.437)		(0.8501-1.55)		
	CC	386	403	1.000		1.000		
	CT	82	103	0.831	0.260	0.899	0.547	0.976
				(0.6024-1.1468)		(0.6350-1.2722)		
	TT	14	2	7.308	0.009	7.250	0.014	0.286
				(1.6501-32.3685)		(1.5021-34.9920)		
	Dom			0.955	0.769	1.022	0.897	0.966
				(0.7001-1.301)		(0.731-1.43)		
	Rec			7.568	0.008	7.462	0.012	0.254
				(1.711-33.48)		(1.554-35.83)		
GNPTG	rs2887538							
	G Allele	713	709	1.000		1.000		
	A Allele	253	309	0.814	0.040	0.850	0.132	0.401
				(0.6689-0.9909)		(0.6879-1.05)		
	GG	265	245	1.000		1.000		
	AG	183	219	0.773	0.054	0.829	0.195	0.775
				(0.5944-1.0041)		(0.6243-1.1006)		
	AA	35	45	0.719	0.173	0.754	0.275	0.481
				(0.4473-1.1559)		(0.4533-1.2525)		
	Dom			0.763	0.034	0.815	0.138	0.454
				(0.5947-0.98)		(0.6225-1.068)		
	Rec			0.806	0.357	0.816	0.421	0.695
				(0.5083-1.277)		(0.4968-1.34)		
NAGPA	rs882294							
	T Allele	785	877	1.000		1.000		
	C Allele	179	143	1.404	0.006	1.531	*0.002*	0.034
				(1.102-1.789)		(1.176-1.994)		
	TT	318	377	1.000		1.000		
	CT	149	123	1.436	0.012	1.577	0.004	0.074
				(1.0837-1.9032)		(1.1609-2.1408)		
	CC	15	10	1.778	0.166	2.060	0.112	0.337
				(0.7880-4.0131)		(0.8443-5.0280)		
	Dom			1.462	0.006	1.611	0.002	0.036
				(1.113-1.921)		(1.197-2.169)		
	Rec			1.606	0.252	1.793	0.195	0.560
				(0.7144-3.61)		(0.742-4.333)		

In *GNPTAB*, we genotyped 11 Tag SNPs and found nominal association of one SNP with dyslexia before adjustment (Additional file 1). SNP rs10778148 showed significant association with dyslexia under recessive model (P = 0.007633, OR = 7.568) and in homozygous genotype (P = 0.008803, OR = 7.3083). After the adjustment for age and sex, the association between SNP rs10778148 and dyslexia remained significant under recessive model (P = 0.01205, OR = 7.462) and in homozygous genotype (P = 0.01364, OR = 7.2499). Moreover, we found rs17031962 achieved significant level under dominant model (P_adjusted_ = 0.003443, OR = 0.6647) and in heterozygous genotype (P_adjusted_ = 0.007001, OR = 0.6738) after adjustment for age and sex. However, only the P-value of rs17031962 under dominant model (P_adjusted_ = 0.0357) remained significant after the FDR adjustment for multiple comparisons.

In *GNPTG*, we genotyped 2 Tag SNPs (Additional file 2) and only found one SNP significantly associated with dyslexia before adjustment. SNP rs2887538 showed significantly associated with dyslexia under dominant model (P = 0.03411, OR = 0.7634). However, no significant association was found after FDR correction.

In *NAGPA*, we genotyped 8 Tag SNPs (Additional file 3) and only found one SNP significantly associated with dyslexia before adjustment. SNP rs882294 showed significantly associated with dyslexia under additive model (P = 0.006043, OR = 1.404), dominant model (P = 0.006426, OR = 1.462) and in heterozygous genotype (P = 0.01175, OR = 1.4361). After the adjustment for age and sex, the association between SNP rs882294 and dyslexia remained significant under additive model (P_adjusted_ = 0.001571, OR = 1.531), dominant model (P_adjusted_ = 0.00167, OR = 1.611) and in heterozygous genotype (P_adjusted_ = 0.003546, OR = 1.6765). While after FDR correction, the association between SNP rs882294 and dyslexia remained significant under additive model (P_adjusted_ = 0.0336) and dominant model (P_adjusted_ = 0.0357).

### Haplotype analysis

**Figure 1:**
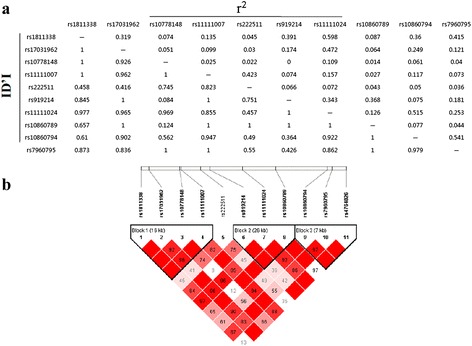
**Linkage disequilibrium analysis of the 11 SNPs in GNPTAB investigated in healthy controls (a).** Three blocks were identified using Haploviewsoftware **(b)**.

**Figure 2:**
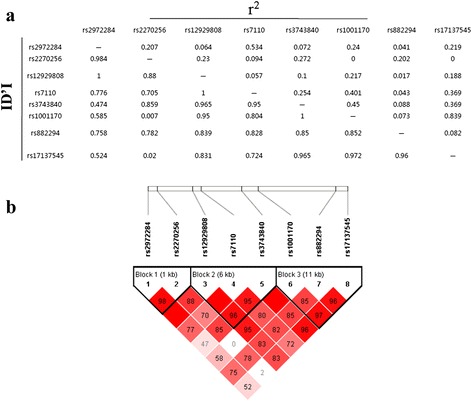
**Linkage disequilibrium analysis of the 8 SNPs in NAGPA investigated in healthy controls (a).** Three blocks were identified using Haploviewsoftware **(b)**.

We built 3 blocks within *GNPTAB* and 3 blocks within *NAGPA* through Haploview software (Figures 1 and 2).

In *GNPTAB*, haplotype analysis was conducted in three blocks (Table 2). All blocks were not associated with dyslexia (P > 0.05 Omnibus test), but a four marker protective haplotype TTCT (Block1 rs1811338-rs17031962-rs10778148-rs11111007) was identified after adjustment for age and sex (P_adjusted_ = 0.00985, OR = 0.761). However, all P-values failed to reach significance after the FDR correction.

**Table 2:** **Haplotypes of the three blocks in GNPTAB between developmental dyslexia and control subjects**

**Haplotype**	**Haplotype frequency**		**OR**	**P**_***unadjusted***_	**OR**	**P**_***adjusted***_	**P**_**FDR**_
	**Patient**	**Control**					
OMNIBUS			NA	0.354	NA	0.078	0.204
TCCC	0.169	0.174	0.965	0.764	1.060	0.660	
TCTT	0.113	0.104	1.090	0.541	1.150	0.372	
TTCT	0.297	0.331	0.853	0.103	0.761	0.010	
GCCT	0.418	0.388	1.130	0.178	1.160	0.128	
OMNIBUS			NA	0.194	NA	0.166	0.212
TCT	0.102	0.113	0.888	0.415	0.932	0.651	
CAC	0.463	0.497	0.880	0.152	0.831	0.055	
TCC	0.177	0.144	1.260	0.056	1.240	0.108	
CCC	0.258	0.246	1.050	0.617	1.130	0.276	
OMNIBUS			NA	0.190	NA	0.177	0.212
CCG	0.134	0.125	1.080	0.558	1.180	0.249	
CTC	0.286	0.254	1.170	0.120	1.150	0.197	
ACC	0.577	0.619	0.850	0.074	0.833	0.063	

In *NAGPA*, haplotype analysis was conducted in three blocks (Table 3). Block 3 consisting of rs1001170, rs882294 and rs17137545 was associated with dyslexia (P = 0.0228 Omnibus test), and included one risk haplotype TCT (P_unadjusted_ = 0.0129, OR = 1.38). After adjustment for age and sex, the association for haplotype TCT in Block 1 remain significant (P_adjusted_ = 0.00289, OR = 1.52), and a risk haplotype GTC in Block 2 (rs12929808-rs7110-rs3743840) achieved significant level (P_adjusted_ = 0.0494, OR = 1.28). However, all P-values failed to reach significance after the FDR correction.

**Table 3:** **Haplotypes of the three blocks in NAGPA between developmental dyslexia and control subjects**

**Haplotype**	**Haplotype frequency**		**OR**	**P**_***unadjusted***_	**OR**	**P**_***adjusted***_	**P**_**FDR**_
	**Patient**	**Control**					
Block1 rs2972284-rs2270256							
OMNIBUS			NA	0.494	NA	0.467	0.467
CC	0.333	0.328	1.030	0.770	1.060	0.572	
TT	0.322	0.302	1.090	0.381	1.080	0.470	
CT	0.344	0.369	0.896	0.253	0.880	0.218	
Block2 rs12929808-rs7110-rs3743840							
OMNIBUS			NA	0.203	NA	0.102	0.204
GTT	0.388	0.411	0.900	0.263	0.881	0.210	
GCC	0.280	0.267	1.070	0.511	1.080	0.493	
ATC	0.108	0.125	0.840	0.217	0.787	0.117	
GTC	0.212	0.183	1.220	0.090	1.280	0.049	
Block3 rs1001170-rs882294-rs17137545							
OMNIBUS			NA	0.078	NA	0.023	0.137
GTC	0.337	0.345	0.965	0.709	0.954	0.648	
TCT	0.170	0.131	1.380	0.013	1.520	0.003	
GTT	0.026	0.027	0.979	0.941	0.896	0.722	
TTT	0.445	0.482	0.859	0.094	0.831	0.061	

## Discussion

Generally, deficits in speech and language functions can be characterized as expressive (production), as receptive (comprehension) or as mixed [4]. Genetically, different mental disorders may share some common factors [1,11-20]. The present study aimed to identify the correlation between dyslexia and three stuttering associated genes, *GNPTAB*, *GNPTG*, and *NAGPA*. Our data showed that genetic variants of *GNPTAB* and *NAGPA* might contribute to the pathogenesis of dyslexia.

*GNPTAB* and *GNPTG* genes encode the alpha and beta subunits and gamma subunit of enzyme UDP-GlcNAc-1-phosphotransferase (GNPT), which is essential to proper trafficking of lysosomal acid hydrolases [25]. Mutations in *GNPTAB* and *GNPTG* genes could cause mucolipidosis types II and III, which are severe forms of autosomal recessive lysosomal storage diseases [26,27]. Here we identified that two SNP markers, rs17031962 and rs10778148, were associated with dyslexia with significant adjusted p-value. However, only an intronic SNP marker rs17031962 was associated with dyslexia under dominant model after the FDR correction.

Moreover, *NAGPA* encodes a Golgi enzyme that catalyzes the second step in the formation of the mannose 6-phosphate recognition marker on lysosomal hydrolases [28]. Our data showed that SNP rs882294 was associated with dyslexia with the allele C as a risk factor after FDR correction. Recently, three mutations in the *NAGPA* gene including one deletion and two missenses have been identified in patients with persistent stuttering. Further biochemical analysis shows that these mutations could impair folding and change degradation activity by the proteasomal system [29]. Since both *GNPTAB* and *NAGPA* are involved in lysosomal decomposition, the above evidence may reveal a potential role for inherited enzyme deficiencies in lysosomal metabolism in speech and language disorders such as stuttering and dyslexia. Furthermore, this knowledge may trigger a variety of new investigations that could help to explore the biological mechanism underlying speech and language disorders.

## Conclusion

In conclusion, we found significant association between development dyslexia and genetic variants in genes encoding the lysosomal targeting system in a large unrelated Chinese cohort. Our data also supported that there are common genetic factors underlying the pathophysiology of different speech and language disorders.

## Methods

### Subjects

Dyslexia screening underwent the two-stage procedures as previously reported. The criteria for dyslexic patients and healthy individuals was described previously [30]. This study was approved by the ethical committee of Tsinghua University School of Medicine. The guardians of children under 16 gave informed, written consent about participation in the study. Briefly, 6,900 primary school students aged between 7 to 13 from Shandong province of China were subjected to a Chinese reading test consisting of character-, word-, and sentence-level questions. Then, 1794 participants whose reading scores were above 87th percentile or below the 13th percentile among all students in the same grade were chosen for further evaluation. These participants were subjected to a character reading test composed of 300 Chinese characters individually for the assessment of reading ability. Then the Raven’s Standard Test was performed to exclude individuals with intelligent deficiency. In total, 1024 children were selected for subsequent analysis, including 502 dyslexic patients and 522 controls.

### SNP markers selection and genotyping

In total, 21 Tag SNPs covering *GNPTAB*, *GNPTG* and *NAGPA* were selected through Tagger program [31] with parameters of minor allele frequency (MAF) over 5% and pairwise r^2^ threshold of 0.8. The SNP genotyping was performed on SequenomMassARRAY platform (Sequenom, San Diego, CA) at CapitalBio Corporation (Beijing, China). Genomic DNA samples were extracted from saliva samples using Oragene™ DNA self-collection kit (DNA Genotek Inc., Ottawa, Ontario, Canada) and DNA quantity was determined by Nanodrop spectrophotometry (Nanodrop 1000 Spectrophotometer, Thermo Scientific, Wilmington, DE). A locus-specific PCR reaction based on a locus-specific primer extension reaction was designed using the MassARRAY Assay Design software package (v3.1). MALDI-TOF mass spectrometer and Mass ARRAY Type 4.0 software were used for mass determination and data acquisition.

### Data analysis

Statistical analysis was undertaken using PLINK software (http://pngu.mgh.harvard.edu/~purcell/plink/), which is an open-source whole genome association analysis toolset and is commonly used to perform a range of basic, large-scale analyses [32]. Hardy-Weinberg equilibrium (HWE) tests were undertaken for each SNP, and association tests were performed using additive, dominant, or recessive genetic models. Haplotype analyses were performed using Haploview software (Version 4.2). Haploview is a software package that provides computation of linkage disequilibrium (LD) in genetic data, performs association studies, chooses tagSNPs and estimates haplotype frequencies [33,34]. Chi square tests were used to test for haplotype association and full model association (Genotype, Dom, Rec). A Fisher’s exact test was used for allelic association. Logistic regression was applied for risk stratification with or without covariate (age and sex) in both single marker and haplotype analysis. False discovery rate (FDR) correction for multiple testing was undertaken for the 21 SNPs that were adopted into the single site association analysis.

## Additional files

Additional file 1: Table S1. Association between SNPs in GNPTAB and dyslexia using the additive, dominant, genotype, and the recessive models. Additional file 2: Table S2. Association between SNPs in GNPTG and dyslexia using the additive, dominant, genotype, and the recessive models. Additional file 3: Table S3. Association between SNPs in NAGPA and dyslexia using the additive, dominant, genotype, and the recessive models.

## Abbreviations

SSD: Speech sound disorder
SLI: Specific language impairment
DD: Developmental dyslexia
RD: Reading disability
GNPTAB: N-acetylglucosamine-1-phosphate transferase gene
GNPTG: N-acetylglucosamine-1-phosphate transferase, gamma subunit
NAGPA: N-acetylglucosamine-1-phosphodiester alpha-N-acetylglucosaminidase
FDR: False discovery rate
MAF: Minor allele frequency
HWE: Hardy-Weinberg equilibrium
LD: Linkage disequilibrium
