# Supplementary material for: Association study of stuttering candidate genes GNPTAB, GNPTG and NAGPA with dyslexia in Chinese population
Source: BMC Genet. 2015 Feb 3;16:7. doi: 10.1186/s12863-015-0172-5 (PMC4342093; doi:10.1186/s12863-015-0172-5)
Supplement: Additional file 1: Table S1. — Association between SNPs in GNPTAB and dyslexia using the additive, dominant, genotype, and the recessive models. [file 12863_2015_172_MOESM1_ESM.docx]

Additional file 1: Table S1. Association between SNPs in GNPTAB and dyslexia using the additive, dominant, genotype, and the recessive models.

| **SNP** | **Patient** | **Control** | **Crude OR** | **unadjusted** | **Adjusted OR** | **adjusted** | **FDR Corrected p-value** |
| --- | --- | --- | --- | --- | --- | --- | --- |
|  |  |  | **(95%CI)** | **p-value** | **(95%CI)** | **p-value** |  |
| rs1811338 |  |  |  |  |  |  |  |
| T Allele | 563 | 622 | 1 |  | 1 |  |  |
| G Allele | 403 | 394 | 1.126 | 0.19 | 1.158 | 0.134 | 0.401 |
|  |  |  | (0.9431-1.344) |  | (0.956-1.402) |  |  |
| TT | 171 | 191 | 1 |  | 1 |  |  |
| GT | 221 | 240 | 1.029 | 0.841 | 1.051 | 0.744 | 0.976 |
|  |  |  | (0.7808-1.3549) |  | (0.7806-1.4141) |  |  |
| GG | 91 | 77 | 1.32 | 0.138 | 1.427 | 0.084 | 0.326 |
|  |  |  | (0.9145-1.9053) |  | (0.9527-2.1387) |  |  |
| Dom |  |  | 1.099 | 0.473 | 1.136 | 0.371 | 0.857 |
|  |  |  | (0.8486-1.424) |  | (0.8589-1.504) |  |  |
| Rec |  |  | 1.299 | 0.123 | 1.358 | 0.096 | 0.56 |
|  |  |  | (0.9315-1.813) |  | (0.9472-1.946) |  |  |
| rs17031962 |  |  |  |  |  |  |  |
| C Allele | 677 | 678 | 1 |  | 1 |  |  |
| T Allele | 287 | 340 | 0.844 | 0.082 | **0.748** | **0.006** | 0.065 |
|  |  |  | (0.6977-1.022) |  | **(0.6079-0.9209)** |  |  |
| CC | 240 | 222 | 1 |  | 1 |  |  |
| CT | 197 | 234 | 0.779 | 0.062 | **0.674** | **0.007** | 0.074 |
|  |  |  | (0.5986-1.0131) |  | **(0.5057-0.8977)** |  |  |
| TT | 45 | 53 | 0.785 | 0.279 | 0.672 | 0.093 | 0.326 |
|  |  |  | (0.5072-1.2161) |  | (0.4226-1.0687) |  |  |
| Dom |  |  | 0.78 | 0.052 | **0.665** | **0.003** | **0.036** |
|  |  |  | (0.6074-1.002) |  | **(0.5056-0.8739)** |  |  |
| Rec |  |  | 0.886 | 0.571 | 0.771 | 0.255 | 0.596 |
|  |  |  | (0.5831-1.346) |  | (0.4917-1.207) |  |  |
| rs10778148 |  |  |  |  |  |  |  |
| C Allele | 854 | 909 | 1 |  | 1 |  |  |
| T Allele | 110 | 107 | 1.09 | 0.54 | 1.148 | 0.368 | 0.639 |
|  |  |  | (0.827-1.437) |  | (0.8501-1.55) |  |  |
| CC | 386 | 403 | 1 |  | 1 |  |  |
| CT | 82 | 103 | 0.831 | 0.26 | 0.899 | 0.547 | 0.976 |
|  |  |  | (0.6024-1.1468) |  | (0.6350-1.2722) |  |  |
| TT | 14 | 2 | **7.308** | **0.009** | **7.25** | **0.014** | 0.286 |
|  |  |  | **(1.6501-32.3685)** |  | **(1.5021-34.9920)** |  |  |
| Dom |  |  | 0.955 | 0.769 | 1.022 | 0.897 | 0.966 |
|  |  |  | (0.7001-1.301) |  | (0.731-1.43) |  |  |
| Rec |  |  | **7.568** | **0.008** | **7.462** | **0.012** | 0.254 |
|  |  |  | **(1.711-33.48)** |  | **(1.554-35.83)** |  |  |
| rs11111007 |  |  |  |  |  |  |  |
| T Allele | 796 | 841 | 1 |  | 1 |  |  |
| C Allele | 166 | 179 | 0.98 | 0.864 | 1.07 | 0.596 | 0.785 |
|  |  |  | (0.7781-1.234) |  | (0.8329-1.375) |  |  |
| TT | 331 | 347 | 1 |  | 1 |  |  |
| CT | 134 | 147 | 0.956 | 0.749 | 1.035 | 0.822 | 0.976 |
|  |  |  | (0.7234-1.2623) |  | (0.7656-1.3999) |  |  |
| CC | 16 | 16 | 1.048 | 0.896 | 1.319 | 0.493 | 0.647 |
|  |  |  | (0.5159-2.1305) |  | (0.5978-2.9092) |  |  |
| Dom |  |  | 0.965 | 0.793 | 1.058 | 0.703 | 0.966 |
|  |  |  | (0.7379-1.261) |  | (0.7911-1.415) |  |  |
| Rec |  |  | 1.062 | 0.866 | 1.274 | 0.536 | 0.703 |
|  |  |  | (0.5252-2.149) |  | (0.5922-2.74) |  |  |
| rs222511 |  |  |  |  |  |  |  |
| A Allele | 728 | 760 | 1 |  | 1 |  |  |
| G Allele | 236 | 258 | 0.954 | 0.655 | 0.992 | 0.945 | 0.992 |
|  |  |  | (0.7773-1.172) |  | (0.7948-1.238) |  |  |
| AA | 275 | 281 | 1 |  | 1 |  |  |
| AG | 178 | 198 | 0.919 | 0.525 | 1.033 | 0.824 | 0.976 |
|  |  |  | (0.7069-1.1937) |  | (0.7781-1.3707) |  |  |
| GG | 29 | 30 | 0.988 | 0.964 | 0.865 | 0.638 | 0.744 |
|  |  |  | (0.5775-1.6896) |  | (0.4734-1.5814) |  |  |
| Dom |  |  | 0.928 | 0.558 | 1.015 | 0.914 | 0.966 |
|  |  |  | (0.7217-1.192) |  | (0.7737-1.332) |  |  |
| Rec |  |  | 1.022 | 0.935 | 0.888 | 0.684 | 0.786 |
|  |  |  | (0.6039-1.73) |  | (0.5023-1.571) |  |  |
| rs919214 |  |  |  |  |  |  |  |
| C Allele | 696 | 758 | 1 |  | 1 |  |  |
| T Allele | 270 | 262 | 1.124 | 0.252 | 1.131 | 0.267 | 0.51 |
|  |  |  | (0.9202-1.373) |  | (0.9103-1.405) |  |  |
| CC | 252 | 278 | 1 |  | 1 |  |  |
| CT | 192 | 202 | 1.049 | 0.722 | 1.024 | 0.868 | 0.976 |
|  |  |  | (0.8077-1.3612) |  | (0.7726-1.3579) |  |  |
| TT | 39 | 30 | 1.434 | 0.162 | 1.536 | 0.129 | 0.338 |
|  |  |  | (0.8650-2.3777) |  | (0.8831-2.6701) |  |  |
| Dom |  |  | 1.098 | 0.461 | 1.089 | 0.537 | 0.877 |
|  |  |  | (0.8559-1.41) |  | (0.8313-1.426) |  |  |
| Rec |  |  | 1.405 | 0.176 | 1.512 | 0.132 | 0.56 |
|  |  |  | (0.8583-2.301) |  | (0.8827-2.589) |  |  |
| rs11111024 |  |  |  |  |  |  |  |
| C Allele | 510 | 511 | 1 |  | 1 |  |  |
| A Allele | 446 | 503 | 0.892 | 0.199 | 0.837 | 0.065 | 0.344 |
|  |  |  | (0.7499-1.062) |  | (0.6933-1.011) |  |  |
| CC | 141 | 133 | 1 |  | 1 |  |  |
| AC | 228 | 245 | 0.878 | 0.391 | NA |  |  |
|  |  |  | (0.6518-1.1823) |  |  |  |  |
| AA | 109 | 129 | 0.797 | 0.201 | 0.69 | 0.054 | 0.326 |
|  |  |  | (0.5627-1.1288) |  | (0.4728-1.0062) |  |  |
| Dom |  |  | 0.85 | 0.253 | 0.783 | 0.114 | 0.454 |
|  |  |  | (0.6431-1.123) |  | (0.5787-1.06) |  |  |
| Rec |  |  | 0.866 | 0.334 | 0.792 | 0.149 | 0.56 |
|  |  |  | (0.646-1.16) |  | (0.5766-1.087) |  |  |
| rs10860789 |  |  |  |  |  |  |  |
| C Allele | 865 | 905 | 1 |  | 1 |  |  |
| T Allele | 99 | 115 | 0.9 | 0.47 | 0.94 | 0.692 | 0.839 |
|  |  |  | (0.6767-1.197) |  | (0.6915-1.277) |  |  |
| CC | 388 | 401 | 1 |  | 1 |  |  |
| CT | 89 | 103 | 0.893 | 0.483 | 0.986 | 0.936 | 0.976 |
|  |  |  | (0.6510-1.2251) |  | (0.7001-1.3889) |  |  |
| TT | 5 | 6 | 0.861 | 0.806 | 0.626 | 0.459 | 0.647 |
|  |  |  | (0.2607-2.8452) |  | (0.1808-2.1646) |  |  |
| Dom |  |  | 0.891 | 0.466 | 0.961 | 0.814 | 0.966 |
|  |  |  | (0.6542-1.214) |  | (0.6875-1.342) |  |  |
| Rec |  |  | 0.881 | 0.834 | 0.628 | 0.463 | 0.695 |
|  |  |  | (0.267-2.904) |  | (0.1812-2.177) |  |  |
| rs10860794 |  |  |  |  |  |  |  |
| A Allele | 561 | 631 | 1 |  | 1 |  |  |
| C Allele | 405 | 385 | 1.177 | 0.072 | 1.211 | 0.05 | 0.344 |
|  |  |  | (0.9855-1.405) |  | (0.9997-1.468) |  |  |
| AA | 166 | 201 | 1 |  | 1 |  |  |
| AC | 229 | 229 | 1.211 | 0.173 | 1.249 | 0.147 | 0.775 |
|  |  |  | (0.9194-1.5946) |  | (0.9250-1.6860) |  |  |
| CC | 88 | 78 | 1.366 | 0.096 | 1.456 | 0.065 | 0.326 |
|  |  |  | (0.9458-1.9731) |  | (0.9772-2.1684) |  |  |
| Dom |  |  | 1.25 | 0.09 | 1.299 | 0.067 | 0.454 |
|  |  |  | (0.9654-1.619) |  | (0.9818-1.72) |  |  |
| Rec |  |  | 1.228 | 0.228 | 1.277 | 0.185 | 0.56 |
|  |  |  | (0.8794-1.715) |  | (0.8896-1.832) |  |  |
| rs7960795 |  |  |  |  |  |  |  |
| C Allele | 672 | 752 | 1 |  | 1 |  |  |
| T Allele | 274 | 258 | 1.182 | 0.095 | 1.149 | 0.199 | 0.465 |
|  |  |  | (0.9712-1.438) |  | (0.9294-1.42) |  |  |
| CC | 243 | 282 | 1 |  | 1 |  |  |
| CT | 186 | 188 | 1.148 | 0.308 | 1.127 | 0.413 | 0.976 |
|  |  |  | (0.8804-1.4974) |  | (0.8466-1.5000) |  |  |
| TT | 44 | 35 | 1.459 | 0.12 | 1.354 | 0.252 | 0.481 |
|  |  |  | (0.9064-2.3481) |  | (0.8057-2.2767) |  |  |
| Dom |  |  | 1.197 | 0.162 | 1.164 | 0.273 | 0.716 |
|  |  |  | (0.9306-1.54) |  | (0.8873-1.527) |  |  |
| Rec |  |  | 1.377 | 0.175 | 1.292 | 0.315 | 0.66 |
|  |  |  | (0.867-2.188) |  | (0.7842-2.129) |  |  |
| rs4764826 |  |  |  |  |  |  |  |
| C Allele | 837 | 893 | 1 |  | 1 |  |  |
| G Allele | 129 | 127 | 1.08 | 0.558 | 1.178 | 0.249 | 0.51 |
|  |  |  | (0.8355-1.395) |  | (0.8918-1.555) |  |  |
| CC | 367 | 392 | 1 |  | 1 |  |  |
| CG | 103 | 109 | 1.009 | 0.952 | 1.05 | 0.771 | 0.976 |
|  |  |  | (0.7443-1.3688) |  | (0.7548-1.4615) |  |  |
| GG | 13 | 9 | 1.543 | 0.324 | 2.275 | 0.085 | 0.326 |
|  |  |  | (0.6518-3.6522) |  | (0.8930-5.7960) |  |  |
| Dom |  |  | 1.05 | 0.744 | 1.125 | 0.469 | 0.877 |
|  |  |  | (0.7832-1.408) |  | (0.8184-1.545) |  |  |
| Rec |  |  | 1.54 | 0.325 | 2.232 | 0.09 | 0.56 |
|  |  |  | (0.6521-3.636) |  | (0.883-5.644) |  |  |
