# Supplementary material for: Association study of stuttering candidate genes GNPTAB, GNPTG and NAGPA with dyslexia in Chinese population
Source: BMC Genet. 2015 Feb 3;16:7. doi: 10.1186/s12863-015-0172-5 (PMC4342093; doi:10.1186/s12863-015-0172-5)
Supplement: Additional file 2: Table S2. — Association between SNPs in GNPTG and dyslexia using the additive, dominant, genotype, and the recessive models. [file 12863_2015_172_MOESM2_ESM.docx]

Additional file 2: Table S2. Association between SNPs in GNPTG and dyslexia using the additive, dominant, genotype, and the recessive models.

| **SNP** | **Patient** | **Control** | **Crude OR** | **unadjusted** | **Adjusted OR** | **adjusted** | **FDR Corrected p-value** |
| --- | --- | --- | --- | --- | --- | --- | --- |
|  |  |  | **(95%CI)** | **p-value** | **(95%CI)** | **p-value** |  |
| rs2294605 |  |  |  |  |  |  |  |
| C Allele | 759 | 789 | 1.000 |  | 1.000 |  |  |
| T Allele | 207 | 225 | 0.955 | 0.677 | 1.000 | 0.997 | 0.997 |
|  |  |  | （0.7682-1.187） |  | （0.7904-1.266） |  |  |
| CC | 294 | 305 | 1.000 |  | 1.000 |  |  |
| CT | 171 | 179 | 0.991 | 0.947 | 0.992 | 0.955 | 0.976 |
|  |  |  | (0.7613-1.2902) |  | (0.7454-1.3198) |  |  |
| TT | 18 | 23 | 0.812 | 0.522 | 1.013 | 0.971 | 0.971 |
|  |  |  | (0.4293-1.5355) |  | (0.5100-2.0119) |  |  |
| Dom |  |  | 0.971 | 0.819 | 0.997 | 0.983 | 0.983 |
|  |  |  | （0.7522-1.253） |  | （0.7567-1.314） |  |  |
| Rec |  |  | 0.815 | 0.523 | 1.022 | 0.950 | 0.971 |
|  |  |  | （0.4339-1.529） |  | (0.5174-2.019) |  |  |
| rs2887538 |  |  |  |  |  |  |  |
| G Allele | 713 | 709 | 1.000 |  | 1.000 |  |  |
| A Allele | 253 | 309 | **0.814** | **0.040** | 0.850 | 0.132 | 0.401 |
|  |  |  | **（0.6689-0.9909）** |  | （0.6879-1.05） |  |  |
| GG | 265 | 245 | 1.000 |  | 1.000 |  |  |
| AG | 183 | 219 | 0.773 | 0.054 | 0.829 | 0.195 | 0.775 |
|  |  |  | (0.5944-1.0041) |  | (0.6243-1.1006) |  |  |
| AA | 35 | 45 | 0.719 | 0.173 | 0.754 | 0.275 | 0.481 |
|  |  |  | (0.4473-1.1559) |  | (0.4533-1.2525) |  |  |
| Dom |  |  | **0.763** | **0.034** | 0.815 | 0.138 | 0.454 |
|  |  |  | **（0.5947-0.98）** |  | （0.6225-1.068） |  |  |
| Rec |  |  | 0.806 | 0.357 | 0.816 | 0.421 | 0.695 |
|  |  |  | (0.5083-1.277) |  | (0.4968-1.34) |  |  |
