# Supplementary material for: Association study of stuttering candidate genes GNPTAB, GNPTG and NAGPA with dyslexia in Chinese population
Source: BMC Genet. 2015 Feb 3;16:7. doi: 10.1186/s12863-015-0172-5 (PMC4342093; doi:10.1186/s12863-015-0172-5)
Supplement: Additional file 3: Table S3. — Association between SNPs in NAGPA and dyslexia using the additive, dominant, genotype, and the recessive models. [file 12863_2015_172_MOESM3_ESM.docx]

Additional file 3: Table S3. Association between SNPs in NAGPA and dyslexia using the additive, dominant, genotype, and the recessive models.

| **SNP** | **Patient** | **Control** | **Crude OR** | **unadjusted** | **Adjusted OR** | **adjusted** | **FDR Corrected p-value** |
| --- | --- | --- | --- | --- | --- | --- | --- |
|  |  |  | **(95%CI)** | **p-value** | **(95%CI)** | **p-value** |  |
| rs2972284 |  |  |  |  |  |  |  |
| C Allele | 653 | 709 | 1.000 |  | 1.000 |  |  |
| T Allele | 313 | 309 | 1.099 | 0.328 | 1.087 | 0.426 | 0.639 |
|  |  |  | (0.9096-1.328) |  | (0.8855-1.334) |  |  |
| CC | 222 | 247 | 1.000 |  | 1.000 |  |  |
| CT | 209 | 215 | 1.082 | 0.559 | 1.116 | 0.449 | 0.976 |
|  |  |  | (0.8315-1.4068) |  | (0.8396-1.4843) |  |  |
| TT | 52 | 47 | 1.231 | 0.348 | 1.135 | 0.594 | 0.734 |
|  |  |  | (0.7975-1.9002) |  | (0.7131-1.8048) |  |  |
| Dom |  |  | 1.108 | 0.419 | 1.121 | 0.408 | 0.857 |
|  |  |  | (0.8637-1.422) |  | (0.8557-1.467) |  |  |
| Rec |  |  | 1.186 | 0.421 | 1.089 | 0.711 | 0.786 |
|  |  |  | (0.7826-1.797) |  | (0.6943-1.707) |  |  |
| rs2270256 |  |  |  |  |  |  |  |
| T Allele | 625 | 668 | 1.000 |  | 1.000 |  |  |
| C Allele | 315 | 328 | 1.028 | 0.781 | 1.059 | 0.598 | 0.785 |
|  |  |  | (0.8459-1.25) |  | (0.8562-1.309) |  |  |
| TT | 202 | 217 | 1.000 |  | 1.000 |  |  |
| CT | 221 | 234 | 1.015 | 0.915 | 1.004 | 0.976 | 0.976 |
|  |  |  | (0.7780-1.3232) |  | (0.7531-1.3397) |  |  |
| CC | 47 | 47 | 1.074 | 0.754 | 1.196 | 0.473 | 0.647 |
|  |  |  | (0.6867-1.6804) |  | (0.7339-1.9476) |  |  |
| Dom |  |  | 1.025 | 0.852 | 1.034 | 0.814 | 0.966 |
|  |  |  | (0.7944-1.321) |  | (0.7846-1.362) |  |  |
| Rec |  |  | 1.066 | 0.768 | 1.198 | 0.448 | 0.695 |
|  |  |  | (0.6967-1.632) |  | (0.7515-1.909) |  |  |
| rs12929808 |  |  |  |  |  |  |  |
| G Allele | 860 | 888 | 1.000 |  | 1.000 |  |  |
| A Allele | 106 | 132 | 0.830 | 0.179 | 0.782 | 0.102 | 0.401 |
|  |  |  | (0.6326-1.089) |  | (0.5825-1.05) |  |  |
| GG | 382 | 388 | 1.000 |  | 1.000 |  |  |
| AG | 96 | 112 | 0.871 | 0.376 | 0.818 | 0.237 | 0.775 |
|  |  |  | (0.6404-1.1835) |  | (0.5872-1.1406) |  |  |
| AA | 5 | 10 | 0.508 | 0.220 | 0.457 | 0.190 | 0.443 |
|  |  |  | (0.1720-1.4996) |  | (0.1420-1.4731) |  |  |
| Dom |  |  | 0.841 | 0.256 | 0.788 | 0.150 | 0.454 |
|  |  |  | (0.6235-1.134) |  | (0.5704-1.09) |  |  |
| Rec |  |  | 0.523 | 0.240 | 0.478 | 0.213 | 0.560 |
|  |  |  | (0.1775-1.541) |  | (0.1491-1.529) |  |  |
| rs7110 |  |  |  |  |  |  |  |
| T Allele | 683 | 736 | 1.000 |  | 1.000 |  |  |
| C Allele | 281 | 282 | 1.075 | 0.473 | 1.095 | 0.403 | 0.639 |
|  |  |  | (0.883-1.308) |  | (0.8854-1.354) |  |  |
| TT | 244 | 262 | 1.000 |  | 1.000 |  |  |
| CT | 195 | 212 | 0.988 | 0.926 | 1.056 | 0.707 | 0.976 |
|  |  |  | (0.7606-1.2825) |  | (0.7960-1.400) |  |  |
| CC | 43 | 35 | 1.319 | 0.257 | 1.264 | 0.373 | 0.603 |
|  |  |  | (0.8171-2.1299) |  | (0.7550-2.1149) |  |  |
| Dom |  |  | 1.035 | 0.789 | 1.087 | 0.543 | 0.877 |
|  |  |  | (0.8064-1.327) |  | (0.8305-1.423) |  |  |
| Rec |  |  | 1.327 | 0.233 | 1.241 | 0.400 | 0.695 |
|  |  |  | (0.8335-2.111) |  | (0.7507-2.05) |  |  |
| rs3743840 |  |  |  |  |  |  |  |
| C Allele | 580 | 583 | 1.000 |  | 1.000 |  |  |
| T Allele | 382 | 431 | 0.885 | 0.194 | 0.873 | 0.182 | 0.465 |
|  |  |  | (0.7355-1.064) |  | (0.7147-1.066) |  |  |
| CC | 169 | 160 | 1.000 |  | 1.000 |  |  |
| CT | 242 | 263 | 0.871 | 0.331 | 0.820 | 0.195 | 0.775 |
|  |  |  | (0.6598-1.1502) |  | (0.6070-1.1075) |  |  |
| TT | 70 | 84 | 0.789 | 0.226 | 0.771 | 0.222 | 0.467 |
|  |  |  | (0.5375-1.1581) |  | (0.5069-1.1712) |  |  |
| Dom |  |  | 0.851 | 0.233 | 0.810 | 0.151 | 0.454 |
|  |  |  | (0.6532-1.109) |  | (0.608-1.08) |  |  |
| Rec |  |  | 0.858 | 0.383 | 0.888 | 0.530 | 0.703 |
|  |  |  | (0.6074-1.211) |  | (0.6125-1.287) |  |  |
| rs1001170 |  |  |  |  |  |  |  |
| T Allele | 595 | 625 | 1.000 |  | 1.000 |  |  |
| G Allele | 365 | 387 | 0.991 | 0.920 | 0.969 | 0.755 | 0.839 |
|  |  |  | (0.8255-1.189) |  | (0.7958-1.18) |  |  |
| TT | 184 | 192 | 1.000 |  | 1.000 |  |  |
| GT | 227 | 241 | 0.983 | 0.901 | 0.935 | 0.651 | 0.976 |
|  |  |  | (0.7492-1.2895) |  | (0.6969-1.2534) |  |  |
| GG | 69 | 73 | 0.986 | 0.944 | 0.965 | 0.866 | 0.909 |
|  |  |  | (0.6703-1.4513) |  | (0.6357-1.4641) |  |  |
| Dom |  |  | 0.984 | 0.900 | 0.943 | 0.678 | 0.966 |
|  |  |  | (0.7607-1.272) |  | (0.7138-1.245) |  |  |
| Rec |  |  | 0.996 | 0.982 | 0.993 | 0.971 | 0.971 |
|  |  |  | (0.6977-1.421) |  | (0.6766-1.457) |  |  |
| rs882294 |  |  |  |  |  |  |  |
| T Allele | 785 | 877 | 1.000 |  | 1.000 |  |  |
| C Allele | 179 | 143 | **1.404** | **0.006** | **1.531** | **0.002** | **0.034** |
|  |  |  | **(1.102-1.789)** |  | **(1.176-1.994)** |  |  |
| TT | 318 | 377 | 1.000 |  | 1.000 |  |  |
| CT | 149 | 123 | **1.436** | **0.012** | **1.577** | **0.004** | 0.074 |
|  |  |  | **(1.0837-1.9032)** |  | **(1.1609-2.1408)** |  |  |
| CC | 15 | 10 | 1.778 | 0.166 | 2.060 | 0.112 | 0.337 |
|  |  |  | (0.7880-4.0131) |  | (0.8443-5.0280) |  |  |
| Dom |  |  | **1.462** | **0.006** | **1.611** | **0.002** | **0.036** |
|  |  |  | **(1.113-1.921)** |  | **(1.197-2.169)** |  |  |
| Rec |  |  | 1.606 | 0.252 | 1.793 | 0.195 | 0.560 |
|  |  |  | (0.7144-3.61) |  | (0.742-4.333) |  |  |
| rs17137545 |  |  |  |  |  |  |  |
| T Allele | 632 | 660 | 1.000 |  | 1.000 |  |  |
| C Allele | 334 | 360 | 0.969 | 0.736 | 0.969 | 0.759 | 0.839 |
|  |  |  | (0.805-1.166) |  | (0.7932-1.184) |  |  |
| TT | 204 | 215 | 1.000 |  | 1.000 |  |  |
| CT | 224 | 230 | 1.026 | 0.847 | 1.005 | 0.973 | 0.976 |
|  |  |  | (0.7870-1.3387) |  | (0.7545-1.3387) |  |  |
| CC | 55 | 65 | 0.892 | 0.581 | 0.920 | 0.712 | 0.786 |
|  |  |  | (0.5936-1.3397) |  | (0.5903-1.4330) |  |  |
| Dom |  |  | 0.997 | 0.980 | 0.986 | 0.920 | 0.966 |
|  |  |  | (0.7748-1.282) |  | (0.7506-1.295) |  |  |
| Rec |  |  | 0.880 | 0.512 | 0.904 | 0.632 | 0.781 |
|  |  |  | (0.5999-1.29) |  | (0.5979-1.367) |  |  |
